# Mapping cerebral amyloid angiopathy susceptibility loci and investigating the efficacy of pTIE2 elevation in Alzheimer's Disease progression using diverse mouse genetic contexts

**DOI:** 10.1002/alz70855_101963

**Published:** 2025-12-23

**Authors:** Olivia J Marola, Jonathan Nyandu Kanyinda, Eliana C Liporace, Gareth R Howell

**Affiliations:** ^1^ The Jackson Laboratory, Bar Harbor, ME, USA; ^2^ Emory University, Atlanta, GA, USA; ^3^ University of Maine, Orono, ME, USA; ^4^ Tufts University Graduate School of Biomedical Sciences, Boston, MA, USA

## Abstract

**Background:**

Cerebral amyloid angiopathy (CAA) co‐occurs with neurodegeneration in Alzheimer's disease (AD). Here, genetic backgrounds with CAA susceptibility (WSB.*APP/PS1*) and resilience (B6.*APP/PS1*) are leveraged to map CAA‐susceptibility loci. Furthermore, blood‐brain barrier (BBB) compromise has been hypothesized to impair amyloid clearance and promote vascular deposition. Endothelial TIE2 deactivation has been shown to weaken BBB integrity in other models and may therefore drive CAA. TIE2 activation (pTIE2) is inhibited by endogenous ANGPT2, which can be genetically reduced to increase pTIE2. This study investigates the efficacy of pTIE2 elevation in preventing CAA in AD.

**Methods:**

To map CAA‐susceptibility loci, B6.*APP/PS1* and WSB mice were crossed, generating BXW.*APP/PS1* (F1) offspring. To determine haplosufficiency of these alleles, CAA was evaluated in BXW.*APP/PS1* mice (*n* =  6/sex). F1 mice were intercrossed to generate heterogeneous F2 progeny (*n* =  50/sex), which were evaluated for CAA. F1 mice were then bred to include *Angpt2* heterozygosity (BXW.*Angpt2^‐/+^APP/PS1*) to evaluate the effects of pTIE2 elevation on AD and CAA progression. Brain sections were evaluated for CAA (X34+ deposits within CD31+, COL4+, and/or αSMA+ vasculature) and neuronal cell (NEUN+) loss using immunohistochemistry, and plasma was analyzed for levels of Aβ42 and Aβ40.

**Results:**

CAA was significantly increased in BXW.*APP/PS1* compared to founder strains at 8 months, and F2 mice exhibited varying CAA severity. BXW.*APP/PS1* and BXW.*Angpt2^‐/+^APP/PS1* mice had significantly elevated plasma Aβ42 and Aβ40 levels compared to WSB.*APP/PS1* and B6.*APP/PS1* mice. However, *Angpt2* heterozygosity did not alter plasma Aβ42 or Aβ40 levels, CAA severity, or NEUN+ cell counts within the cortex or hippocampus.

**Conclusions:**

The robust CAA observed in BXW.*APP/PS1* suggests that heterozygous loci promote CAA. The variability in CAA severity among F2 mice indicates that CAA‐susceptibility loci are likely mappable. F2 SNP analysis and R/qtl2 software are being used to identify candidate loci associated with CAA. While pTIE2 elevation did not appear to affect CAA pathogenesis, candidate alleles from our F2 mapping study will be tested to determine whether CAA can be attenuated through genetic and pharmacological interventions.

